# Kruppel-Like Factor 2-Mediated Suppression of MicroRNA-155 Reduces the Proinflammatory Activation of Macrophages

**DOI:** 10.1371/journal.pone.0139060

**Published:** 2015-09-25

**Authors:** Shaolin He, Liyuan Yang, Dazhu Li, Ming Li

**Affiliations:** 1 Department of Cardiology, Institute of Cardiovascular Diseases, Union Hospital Tongji Medical College, Huazhong University of Science and Technology, Wuhan, PR China; 2 Department of Cardiology, XiangYang First People's Hospital Affiliated of HuBei University of Medicine, Hubei, PR China; University of Padua, ITALY

## Abstract

**Objective:**

Recent evidence indicates that significant interactions exist between Kruppel-like factor 2 (KLF2) and microRNAs (miRNAs) in endothelial cells. Because KLF2 is known to exert anti-inflammatory effects and inhibit the pro-inflammatory activation of monocytes, we sought to identify how inflammation-associated miR-155 is regulated by KLF2 in macrophages.

**Approach and Results:**

Peritoneal macrophages from wild-type (WT) C57Bl/6 mice were transfected with either recombinant adenovirus vector expressing KLF2 (Ad-KLF2) or siRNA targeting KLF2 (KLF2-siRNA) for 24 h–48 h, then stimulated with oxidized low-density lipoproteins (ox-LDL, 50 μg/mL) for 24 h. Quantitative real-time polymerase chain reaction showed that KLF2 markedly reduced the expression of miR-155 in quiescent/ox-LDL-stimulated macrophages. We also found that the increased expression of miR-155, monocyte chemoattractant protein (MCP-1) and interleukin (IL)-6 and the decreased expression of the suppressor of cytokine signaling (SOCS)-1 and IL-10 in ox-LDL-treated macrophages were significantly suppressed by KLF2. Most importantly, over-expression of miR-155 could partly reverse the suppressive effects of KLF2 on the inflammatory response of macrophages. Conversely, the suppression of miR-155 in KLF2 knockdown macrophages significantly overcame the pro-inflammatory properties associated with KLF2 knockdown. Finally, Ad-KLF2 significantly attenuated the diet-induced formation of atherosclerotic lesions in apolipoprotein E-deficient (apoE^-/-^) mice, which was associated with a significantly reduced expression of miR-155 and its relative inflammatory cytokine genes in the aortic arch and in macrophages.

**Conclusion:**

KLF2-mediated suppression of miR-155 reduced the inflammatory response of macrophages.

## Introduction

Inflammation is crucial for the initiation and progression of atherosclerosis from the initial lesions to end-stage complications. Macrophage activation exacerbates the inflammatory responses in atheromatous plaques and promotes their structural instability [1]. The inflammatory response could therefore be a critical target in atheromatous lesions to prevent atherogenesis [2]. In recent years, it has become clear that Kruppel-like factor 2 (KLF2) is a central regulator of endothelial and monocyte/macrophage proinflammatory action [3, 4]. Although the effects of KLF2 in macrophage activation predicts that it likely inhibits vascular inflammation, the mechanisms of action of KLF2 in this process remain uncertain.

MiRNAs are small (22 nucleotide long) single-stranded non-coding RNAs transcribed in the nucleus, processed by the enzymes Drosha (DROSHA) and Dicer (DICER1) and incorporated in RNA-induced silencing complexes that mediate the translational inhibition or degradation of target messenger RNAs [5]. Many miRNAs have been identified that play key roles in physiological and pathophysiological processes, including atherosclerosis [6, 7]. MiR-155, a typical multi-functional miRNA, is emerging as a novel regulator involved in the inflammation signaling pathway in the pathogenesis of atherosclerosis. In macrophages, several miRNAs, including miR-155, miR-146, miR-125b, have been found to be substantially up-regulated by Toll-like receptor (TLR) ligands [8, 9]. Although the functional relevance of macrophage miR-155 expression is unclear, studies have indicated that miR-155 shows both anti- and pro-inflammatory effects by regulating TAB2 and SOCS-1, respectively [10, 11, 12]. However, the role of miR-155 in the pathogenesis of atherosclerosis remains unclear. Indeed, two recent studies have shown opposite results regarding the effects of bone marrow cells with miR-155 deficiency on the process of atherosclerosis. One report showed that bone marrow cells with miR-155 deficiency increased atherosclerosis in low-density lipoprotein receptor (LDLR)^−/−^ mice fed a high-fat diet by generating a more pro-atherogenic immune cell profile and a more pro-inflammatory monocyte/macrophage phenotype, indicating that miR-155 is atheroprotective in that model[13] whereas another report showed that miR-155 promoted atherosclerosis in apoE^-/-^ mice by repressing B-cell lymphoma 6 protein in macrophages, thus enhancing vascular inflammation, suggesting that miR-155 is proatherogenic [14].

Given that both KLF2 and miR-155 play key roles in regulating the function of macrophages in the activation of inflammation, we sought to investigate how miR-155 is regulated by KLF2 and might be responsible for mediating the suppression of the pro-inflammatory activation of macrophages by KLF2.

## Materials and Methods

### Recombinant adenoviral KLF2 over-expression

Experiments in which stable recombinant adenoviral KLF2 was over- expressed were performed by constructing recombinant adenoviral vectors expressing KLF2. The entire mouse KLF2 gene open reading frame was obtained by RT-PCR, cloned into the CMV-MCS-EGFP GV135 vector, and ligated into a shuttle plasmid. Subsequently, the shuttle plasmid and adenoviral backbone plasmid were co-transfected into HEK-293A cells to produce the recombinant adenoviral vector Ad-KLF2. An otherwise identical vector without KLF2 cDNA was used to generate empty viruses as controls (EV).

### Preparation of LDL and copper-oxidized LDL

Blood samples for the isolation of lipoproteins were collected in EDTA (1 mg/mL) tubes from lipidemic donors after 12 hours of fasting. After density adjustment with KBr, LDLs (density = 1.03 to 1.063 g/L) were isolated from the plasma by preparative ultracentrifugation at 50 000 rpm for 22 hours using a type 50 rotor as our previously described [15]. LDLs were dialyzed against phosphate-buffered saline (PBS) containing 0.3 mM EDTA, sterilized by filtration through a 0.22-μm filter, and stored under nitrogen gas at 4°C. The protein content was determined by the method of Lowry et al. Copper oxidation of LDL was performed by incubation of post-dialyzed LDL (1 mg of protein/mL in EDTA-free PBS) with copper sulfate (10 mM) for 24 hours at 37°C. Lipoprotein oxidation was confirmed by analysis of thiobarbituric acid-reactive substances.

### Macrophage culture, transfection, siRNA-mediated gene knockdown, and adenovirus infection

Elicited peritoneal macrophages were collected from mice after an intraperitoneal injection of 1 mL of 3% thioglycolate (Sigma-Aldrich). Cells were resuspended in DMEM culture media supplemented with 5% FBS (Atlanta Biologicals) and plated at a concentration of 5 x 10^5^ cells/mL on 10-cm culture plates for 18 hours. Cells were then detached, counted, and re-plated at the required cell density for further treatment. Treated macrophages were either lysed with TRIzol reagent for RNA extraction and RT-PCR or lysed with RIPA buffer containing 1% proteinase inhibitor and 1% phosphatase inhibitor cocktail for Western blotting.

The miRNA mimics and miRNA inhibitors for miR-155 were obtained from Ambion. Peritoneal macrophages were transfected with 30 pmol of miRNA mimic or 50 pmol of miRNA inhibitor using Lipofectamine RNAiMAX reagent (Invitrogen) according to the manufacturer’s instructions. Unless stated otherwise, the cells were incubated for 24 hours post-transfection and then exposed to ox-LDL (50 μg/mL) for another 24 hours. Mouse KLF2-directed siRNA (5’-GCACGGAUGAGGACCUAAAUU-3’) (siRNA-KLF2) and a non-specific control siRNA(5’-CUGGCGCCUUCGGUCU UUUU-3’) (NS) were purchased from Qiagen (Valencia, CA). For siRNA-mediated gene silencing, macrophages were transfected with siRNA duplexes using Lipofect-amine RNAiMAX reagent. Macrophages were transfected with adenovirus vectors at the appropriate multiplicity of infection.

### Mouse treatment and specimen collection

Male apoE^-/-^ mice at five weeks of age were purchased from the Department of Experimental Animals of the Medical Center, Peking University, and were fed a high-fat diet (HFD, Western Diet, TD.88137, Harlan Teklad, Madison, WI) containing 17.3% protein, 48.5% carbohydrate, 21.2% fat, and 0.2% cholesterol by weight, with 42% kcal from fat. C57Bl/6 wild type (WT) control mice were fed a normal chow diet (Oriental Yeast). The animals were bred and maintained in a specific pathogen-free (SPF) barrier facility at Tongji Medical College (Wuhan, China) with a 12 hours light-dark cycle. All animal experiments were approved by the Institutional Animal Care and Use Committee of Tongji Medical College. The protocol was approved by the Ethics Committee of Tongji Medical College, Huazhong University of Science and Technology (IORG No: IORG0003571). All surgery was performed under sodium pentobarbital anesthesia, and all efforts were made to minimize suffering. After 1 weeks of accommodation, the mice were randomly divided into the following four groups: WT control mice treated with EV (WT+EV), WT control mice treated with Ad-KLF2 (WT+Ad-KLF2), apoE-/- mice fed a HFD and treated with EV (apoE-/- +HFD+EV), and apoE-/- mice fed a HFD and treated with Ad-KLF2 (apoE-/-+HFD+Ad-KLF2). The initial concentration of Ad-KLF2 and EV-control vectors was 5×10^11^ plaque-forming units (pfu)/mL. They were diluted in sterile PBS and administered to the mice through a single bolus injection via the jugular vein. Animals were anesthetized with avertin during the vectors injection. After 18 weeks of an atherogenic diet, mice were anesthetized and euthanized, and their aortas were harvested for analysis.

### Atherosclerotic lesion analysis

The arch portions of the aorta were removed and perfused with PBS and snap-frozen in optimal cutting temperature (OCT) medium (Tissue-Tek). To measure the plaque size, longitudinal sections of the aortic arches were examined under the microscope. The composition of the atherosclerotic lesions was analyzed on cryosections of aortic arch tissues fixed in acetone, air-dried, and stained with hematoxylin–eosin (H&E). Total plaque lesion area in the ostia of the innominate, common carotid, and the subclavian arteries of each mouse were used to compute averages per group [16, 17, 18]. Quantification of the atherosclerotic areas was performed by using computer-assisted image quantification (Image-Pro Plus software, Media Cybernetics).

### Quantitative real-time PCR

Total RNA was isolated from cultured macrophages and murine aortic arches using TRIzol reagent (Invitrogen, Carlsbad, CA) and was reverse transcribed using a Taqman miRNA RT kit or high-capacity cDNA reverse transcription kit. MiRNA RT-PCR was performed using Taqman miRNA assays and Taqman universal PCR master mix (Life Technologies). QRT-PCR for mRNAs was performed using gene-specific primers and SYBR Green master mix (Fermentas). All of the real-time PCR experiments were run on a 7900HT RT-PCR system. The data were normalized to endogenous control reference genes (GAPDH for mRNA and U6 for miRNA). Quantitative miRNA expression data were analyzed using an ABI Prism 7900HT Sequence Detection System (Applied Biosystems, Foster City, CA). The expression of miR-155 was detected according to the manufacturer’s instructions using the miScript PCR System (Qiagen, Valencia, CA) and miScript Primer Assays (Cat# MS00001701 for murine miR-155). The following primers were used: mouse KLF2 (forward 5'-ACCAAGAGCTCGCACCTAAA-3' and reverse 5'- GTGGCACTGAA AGGGTCTGT-3'); mouse SOCS-1 (forward 5'-CTGGGATGCCGTGTTATTTT-3' and reverse 5'-TAGGAGGTGCGAG TTCAGGT-3'); mouse MCP-1 (forward 5'-CTCATAGCAGCCACCTTCATTC-3' and reverse 5'-CAAGTCTTCGGAGTTT GGGTTT-3'); mouse IL-6 (forward 5'-CATGTTCTCTGGGAAATCGTGG-3' and reverse 5'-AACGCACTAGGTTTGCCGAGTA-3'); mouse IL-10 (forward 5'-TCAA ACAAAGGACCAGCTGGACAACATACTGC-3' and reverse 5'- CTGTCTAGGT CCTGGAGTCCAGCAGACTCAA-3') and GAPDH (forward 5'-CCACCCATG GCAAATTCCATGGCA-3' and reverse 5'-TCTAGACCGCAGGTCAGGTCCACC- 3').

### Western blot analysis

Proteins were extracted from aortic arch samples, and cell lysate proteins were separated by SDS-polyacrylamide gel electrophoresis and transferred onto PVDF membranes (Amersham Pharmacia Biotech, Uppsala, Sweden). After incubation with the primary antibodies, the membranes were incubated with a peroxidase-conjugated secondary antibody, and the signals were visualized using a chemiluminescence kit (Amersham Pharmacia Biotech). The bands were scanned and quantified using Kodak 1D Image Analysis software. The protein levels were normalized to β-actin and plotted as indicated.

### ELISAs for cytokines

The peritoneal macrophages were collected, and transfection experiments were performed as described in the previous section. Culture supernatants were assayed for the cytokine levels of MCP-1, IL-6, and IL-10 by ELISA according to the manufacturer’s instructions. Each sample was tested for each cytokine in triplicate.

### Statistical analysis

All of the statistical analyses were carried out using the SPSS version 11 software. Unless stated otherwise, the means (+/- SD) of at least 3 independent experiments are shown. Statistical evaluations were carried out using analysis of variance with Tukey’s post-hoc test. P<0.05 was considered statistically significant.

## Results

### Ox-LDL induced miR-155/SOCS-1 and the pro-inflammatory responses of macrophages are regulated by KLF2

**Fig 1:**
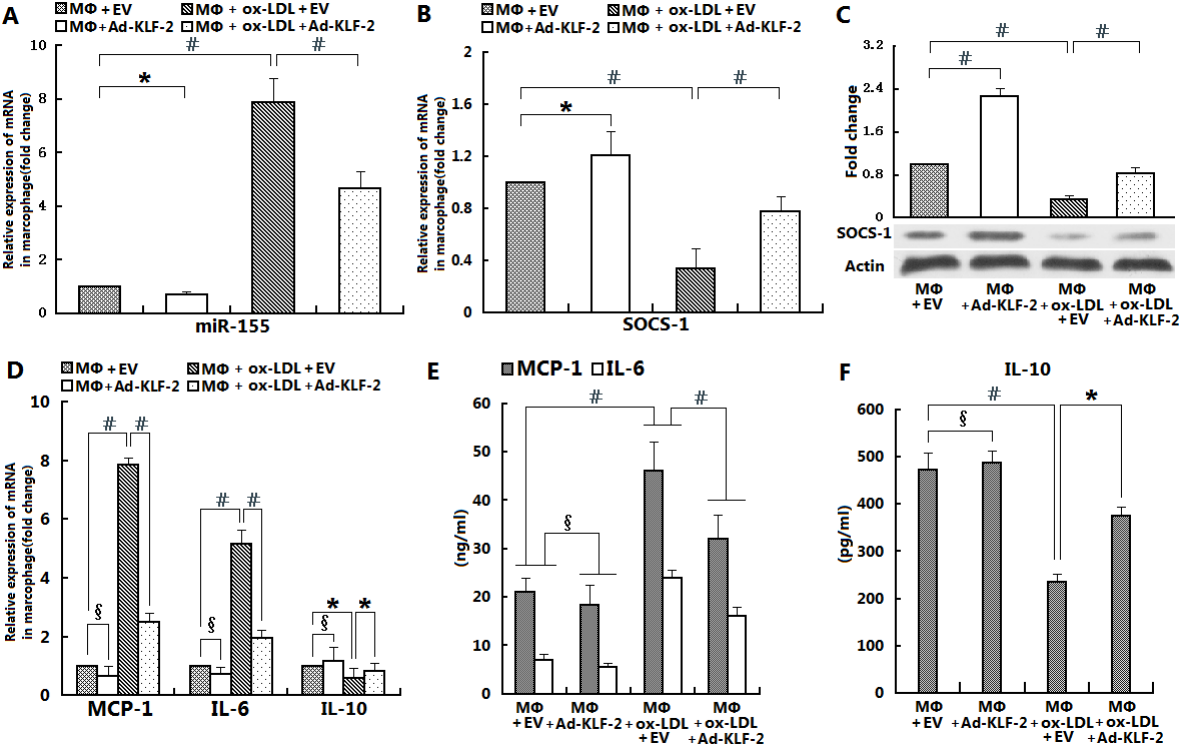
Ox-LDL-induced miR-155/SOCS-1 and pro-inflammatory responses in macrophages are regulated by KLF2 over-expression. The effects of KLF2 over-expression on miR-155 expression in macrophages (mø) cultured alone or activated by ox-LDL were examined. Mø from WT C57BL/6 mice were infected with either empty virus (EV) (as a control) or virus containing Ad-KLF2 for 24–48 h and then stimulated with ox-LDL (50 μg/ml) for 24 h. Total RNA was extracted and subjected to RT-PCR analysis to determine miR-155 mRNA expression levels. Bar graphs indicate relative miR-155 mRNA levels normalized to U6 mRNA levels (A). The effects of KLF2 over-expression on SOCS-1 mRNA (B) and protein (C) expression in Mø cultured alone or activated by ox-LDL were assessed. Data were normalized to an endogenous internal control gene (GAPDH for mRNA and β-actin for protein). The effects of KLF2 over-expression on MCP-1, IL-6, and IL-10 mRNA expression in Mø cultured alone or activated by ox-LDL were examined. Bar graphs depict relative MCP-1, IL-6, and IL-10 mRNA levels normalized to GAPDH mRNA levels (D). Culture supernatants were assessed by ELISA to determine levels of the cytokines MCP-1, IL-6, and IL-10 (E and F). All of the above data are presented as means ± S.D. for three independent experiments (with #, *, and δ indicating p<0.001, p<0.05, and p>0.05, respectively, for the indicated comparisons).

Macrophages respond to various inflammatory stimuli through the differential regulation of a small set of miRNAs and increased expression of pro-inflammatory factors and cytokines/chemokines [8]. It has been reported that SOCS-1, a negative regulator of the TLR4-mediated inflammation pathway, is a miR-155 target protein. Because KLF2 is known to exert anti-inflammatory effects, we undertook over-expression studies to gain insight into the role of KLF2 in the regulation of miR-155 and the expression of its targeting gene SOCS-1 in macrophages. Macrophages from WT C57Bl/6 mice transfected with either EV or Ad-KLF2 for 24 h–48 h were then stimulated with ox-LDL (50 μg/mL) for 24 h. RT-PCR was performed to quantify the levels of miR-155, SOCS-1 and the cytokines MCP-1, IL-6, and IL-10. The levels of SOCS-1 protein and cytokines were also measured by Western blot and ELISA, respectively. The results show that KLF2 markedly reduced the expression of miR-155 in unstimulated macrophages (Fig 1A, p<0.05). We also showed that the exposure of macrophages to ox-LDL led to marked up-regulation of miR-155 but down-regulation of SOCS-1 (Fig 1B and 1C, p<0.05). Most importantly, the ox-LDL-induced increase in miR-155 decreased the expression of SOCS-1 protein associated with a rise in MCP-1 and IL-6 and a decline in IL-10 in macrophages that were significantly suppressed by KLF2 (Fig 1D–1F, # p<0.001 and * p<0.05 for the indicated comparisons).

**Fig 2:**
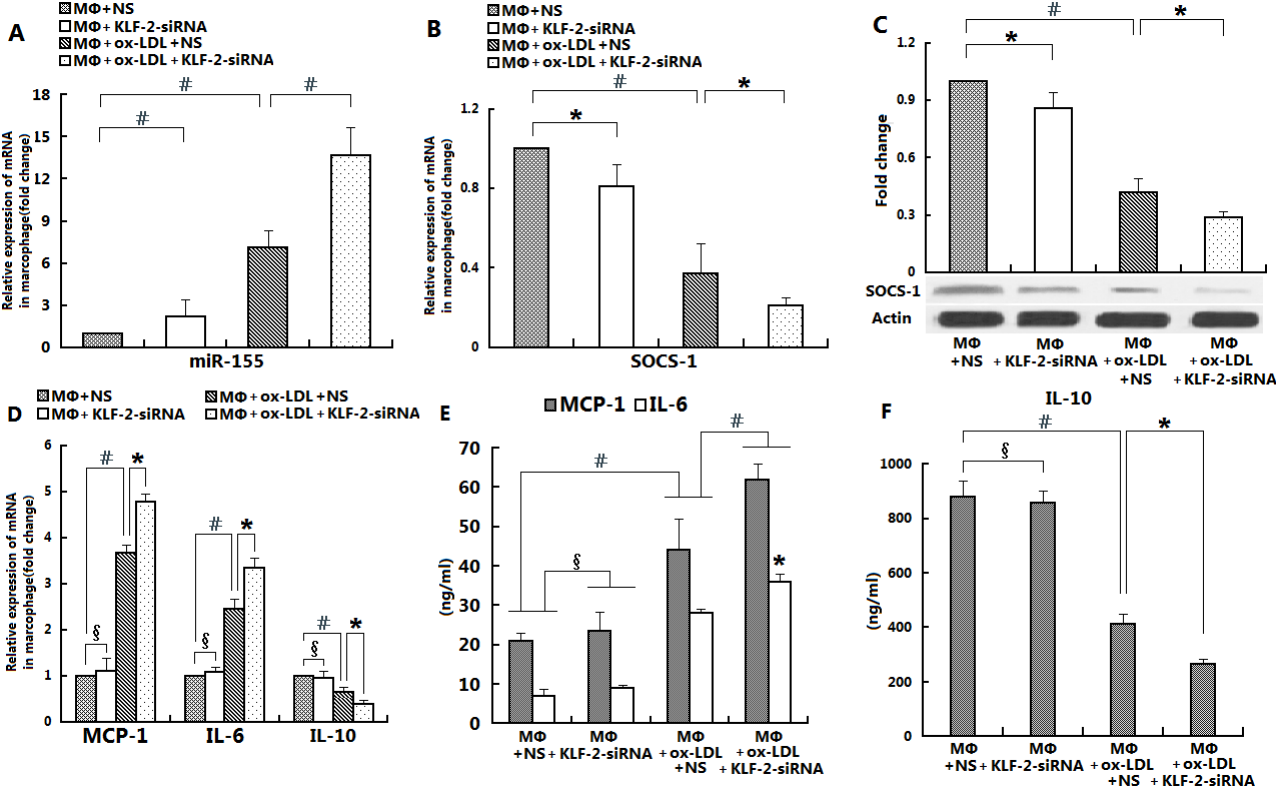
Ox-LDL-induced miR-155/SOCS-1 and pro-inflammatory responses in macrophages are regulated by the silencing of KLF2. The effects of silencing KLF2 on miR-155 expression in macrophages cultured alone or activated by ox-LDL were examined. Macrophages from WT C57BL/6 mice were infected with either viruses containing a nonspecific control siRNA (NS) or viruses containing siRNA-KLF2 for 24–48 h and then stimulated with ox-LDL (50 μg/ml) for 24 h. Total RNA was extracted and subjected to RT-PCR analysis to determine miR-155 mRNA expression levels. Bar graphs indicate relative miR-155 mRNA levels normalized to U6 mRNA levels (A). The effects of silencing KLF2 on SOCS-1 mRNA (B) and protein (C) expression levels in macrophages cultured alone or activated by ox-LDL were assessed. Data were normalized to an endogenous internal control reference gene (GAPDH for mRNA and β-actin for protein). The effects of silencing KLF2 on MCP-1, IL-6, and IL-10 mRNA expression in Mø cultured alone or activated by ox-LDL were examined. Bar graphs depict relative MCP-1, IL-6, and IL-10 mRNA levels normalized to GAPDH mRNA levels (D). Culture supernatants were evaluated by ELISA to determine levels of the cytokines MCP-1, IL-6, and IL-10 (E and F). All of the above data are presented as means ± S.D. for three independent experiments (with #, *, and δ indicating p<0.001, p<0.05, and p>0.05, respectively, for the indicated comparisons).

To further confirm the regulation of macrophage miR-155, its targeting gene SOCS-1 and inflammatory cytokines by KLF2, small interfering RNA-mediated (siRNA-KLF2) knockdown studies were also undertaken. Consistent with the results of our over-expression studies, our data showed that miR-155 was increased by KLF2 knockdown in un-stimulated and ox-LDL-stimulated macrophages (Fig 2A, p<0.05). KLF2 knockdown increased ox-LDL-induced pro-inflammatory activation of macrophages by increasing miR-155 and decreasing SOCS-1 expression (Fig 2B–2F). By using both gain-of-function and loss-of-function approaches, our above data strongly suggest that miR-155 and its targeted gene SOCS-1 expression in macrophages are regulated by KLF2 and are correlated with the expression of inflammatory cytokines.

### Role of miR-155 in KLF2-mediated inhibition of macrophage activation

**Fig 3:**
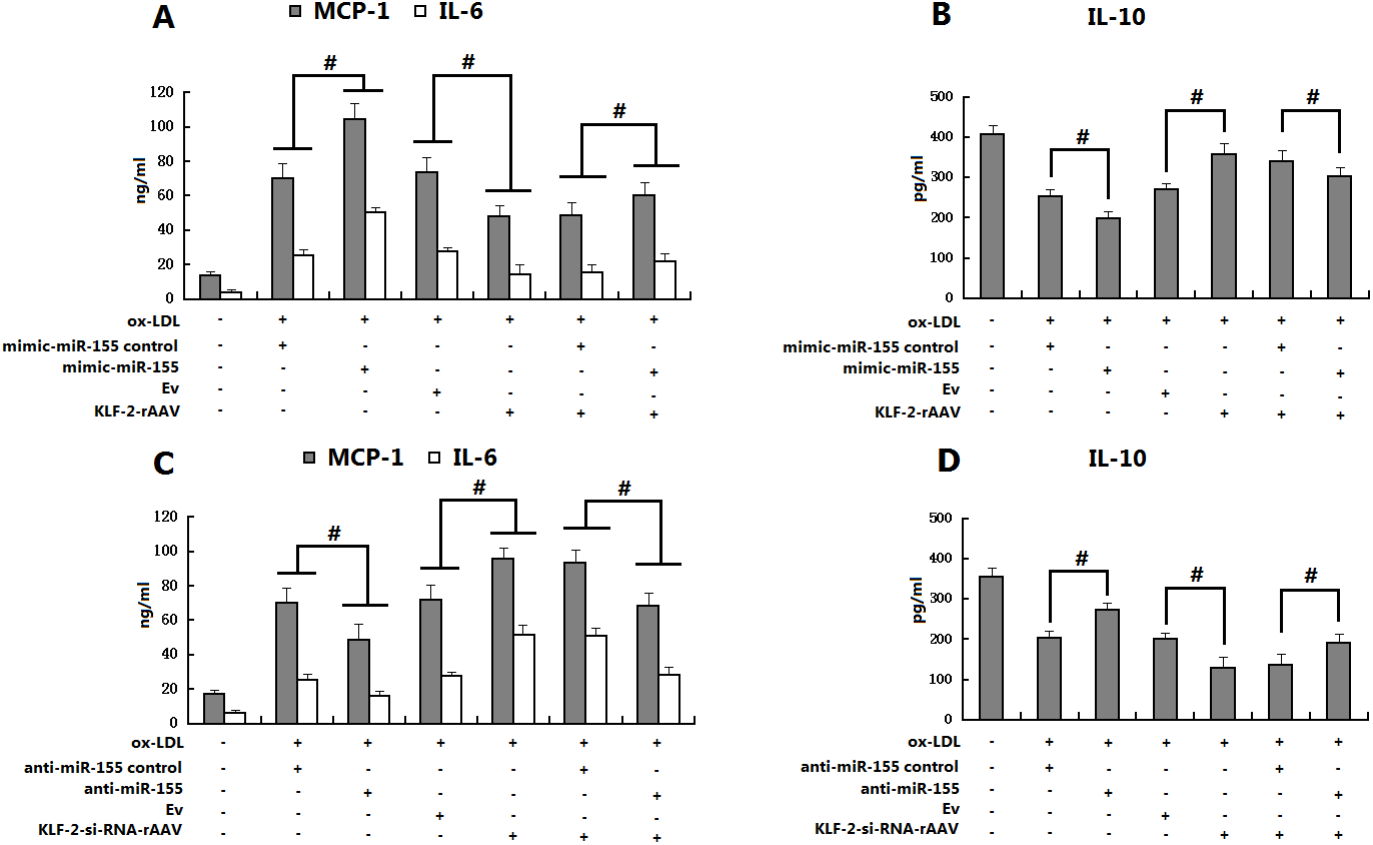
Role of miR-155 in the KLF2-induced inhibition of macrophage activation. To further confirm the role of miR155 in the KLF2-induced inhibition of macrophage inflammatory responses, WT macrophages and macrophages infected with EV or with viruses containing Ad-KLF2, siRNA-KLF2, or nonspecific control siRNA (NS) were cultured for 24 h. Using Lipofectamine 2000, mimic-miR-155 or anti-miR-155 was transfected into macrophages. Unless otherwise stated, cells were incubated for 24 hours post-transfection and then exposed to ox-LDL (50 μg/ml) for an additional 24 hours. Culture supernatants were evaluated by ELISA to determine MCP-1 and IL-6 levels. Data are presented as means ± S.D. for three independent experiments. The effects of miR-155 over-expression on MCP-1 and IL-6 expression in ox-LDL-stimulated macrophages were examined (A). The impact of miR-155 over-expression on IL-10 expression in ox-LDL-stimulated macrophages was evaluated (B). The effects of silencing miR-155 on MCP-1 and IL-6 expression in ox-LDL stimulated macrophages were assessed (C). The impact of silencing miR-155 on IL-10 expression in ox-LDL stimulated macrophages was examined (D) (# and δ indicate p<0.05 and p>0.05, respectively, for the indicated comparisons).

To demonstrate the functional relationship between miR-155 and KLF2, mimic- miR-155 or mimic-miR-155 control was transfected into KLF2 over-expressing peritoneal macrophages using Lipofectamine 2000. Unless otherwise stated, the cells were incubated for 24 hours post-transfection and then exposed to ox-LDL (50 μg/mL) for another 24 hours. The production of cytokines in macrophages was determined by ELISA. Our results showed that increased levels of MCP-1 and IL-6 in macrophages stimulated by ox-LDL were further enhanced by the over-expression of miR-155. Most interestingly, restoration of miR-155 levels in KLF2 over-expressing macrophages increased MCP-1 and IL-6 but reduced IL-10 expression, indicating that the over-expression of miR-155 could partly reverse the suppressing effects of KLF2 on the macrophage inflammatory response induced by ox-LDL (Fig 3A and 3B, # p<0.05 for the indicated comparisons). To further confirm a possible role of miR-155 in the KLF2-mediated inhibition of macrophage inflammatory responses, we transfected anti-miR-155 (inhibitor) into KLF2 knockdown macrophages and exposed the transfected cells to ox-LDL after 24 hours. The anti-miR-155 transfected cells showed that the increased levels of MCP-1 and IL-6 but decreased levels of IL-10 induced by ox-LDL were significantly reduced compared with the miRNA control transfected cells. The suppression of miR-155 activity in KLF2 knockdown macrophages with anti-miR-155 significantly overcame the pro-inflammatory properties associated with KLF2 knockdown (Fig 3C and 3D, # p<0.05 for the indicated comparisons).

### The effect of KLF2 on miR-155/SOCS-1, cytokine gene expression and atherosclerotic lesion formation in the aortic arches of apoE-deficient mice

**Fig 4:**
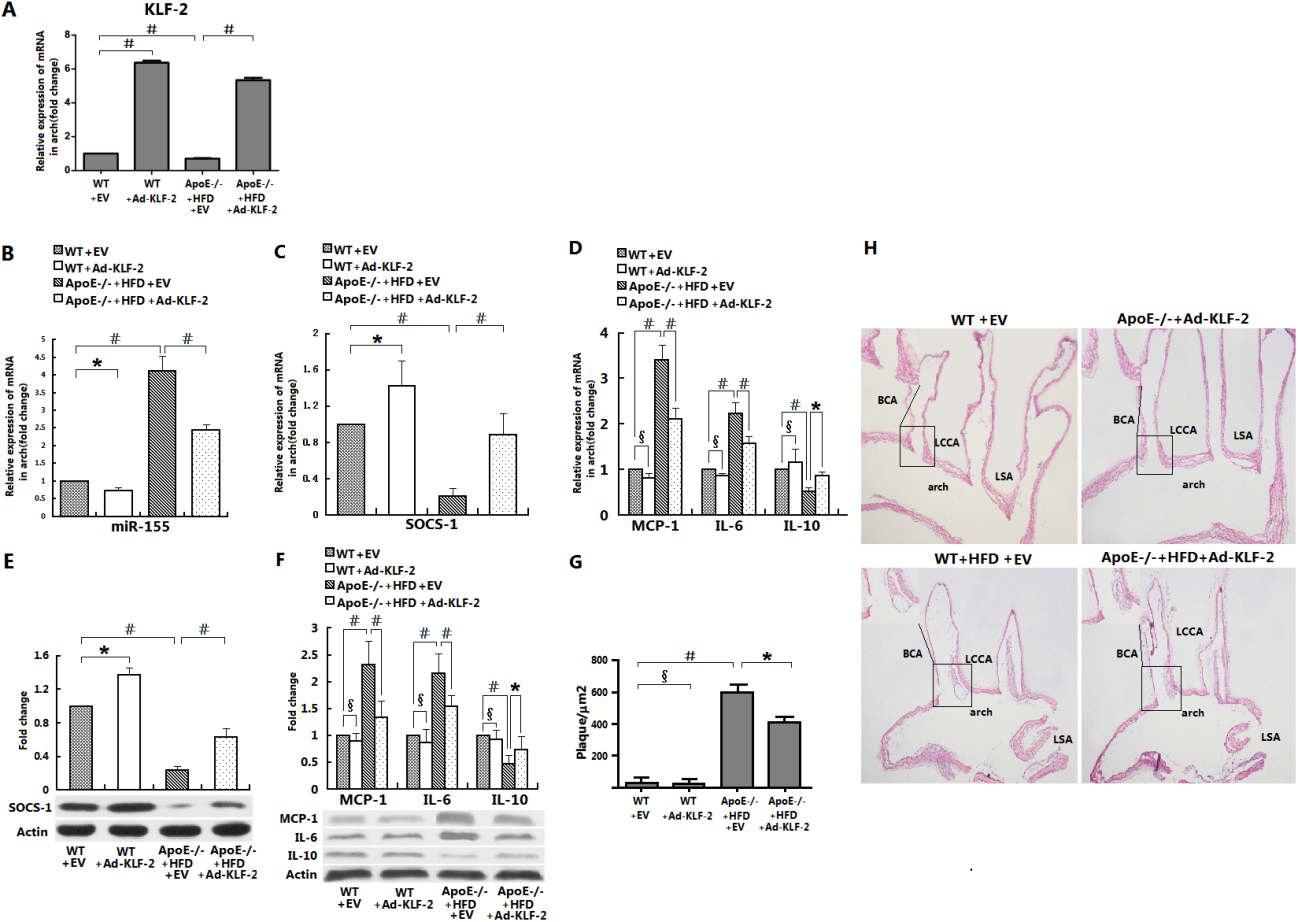
Effects of KLF2 on miR-155/SOCS-1 and cytokine gene expression and the formation of atherosclerotic lesions in the aortic arches of apoE-deficient mice. Five-week-old male littermate apoE^-/-^ mice and WT controls were fed a high-fat, high-cholesterol diet (HFD) and a normal diet, respectively. Mice were randomly divided into the following four groups: WT+EV, WT+Ad-KLF2, apoE^-/-^+HFD+EV, and apoE^-/-^+HFD+Ad-KLF2. After 18 weeks on an atherogenic diet, the mice were euthanized, and the arch portions of their aortas were harvested for analysis. KLF2, miR-155, and SOCS-1 expression in the aortic arch were analyzed by RT-PCR, and the areas of atherosclerotic lesions in aortic arch regions were detected by hematoxylin–eosin staining. Bar graphs indicate relative mRNA levels for KLF2 normalized to GAPDH mRNA levels (A); miR-155 normalized to U6 mRNA levels (B); SOCS-1 normalized to GAPDH mRNA levels (C); and cytokine genes (MCP-1, IL-6, and IL-10) normalized to GAPDH mRNA levels (D). SOCS-1 (E) and cytokine (F) protein expression in the aortic arch were detected by Western blotting. Treatment with Ad-KLF2 was associated with a reduction in aortic arch atherosclerosis among apoE^-/-^ mice fed an HFD. The brachiocephalic trunk artery (BCA), left common carotid artery (LCCA) and left subclavian artery (LSA) are indicated in the figure (H). Ad-KLF2 treatment produced a reduction in the size of atherosclerotic lesions. Photomicrographs of longitudinal sections of the mouse aortic arch-wall region were analyzed by computer-assisted image quantification (G) (#, *, and δ indicate p<0.001, p<0.05, and p>0.05, respectively, for the indicated comparisons).

**Fig 5:**
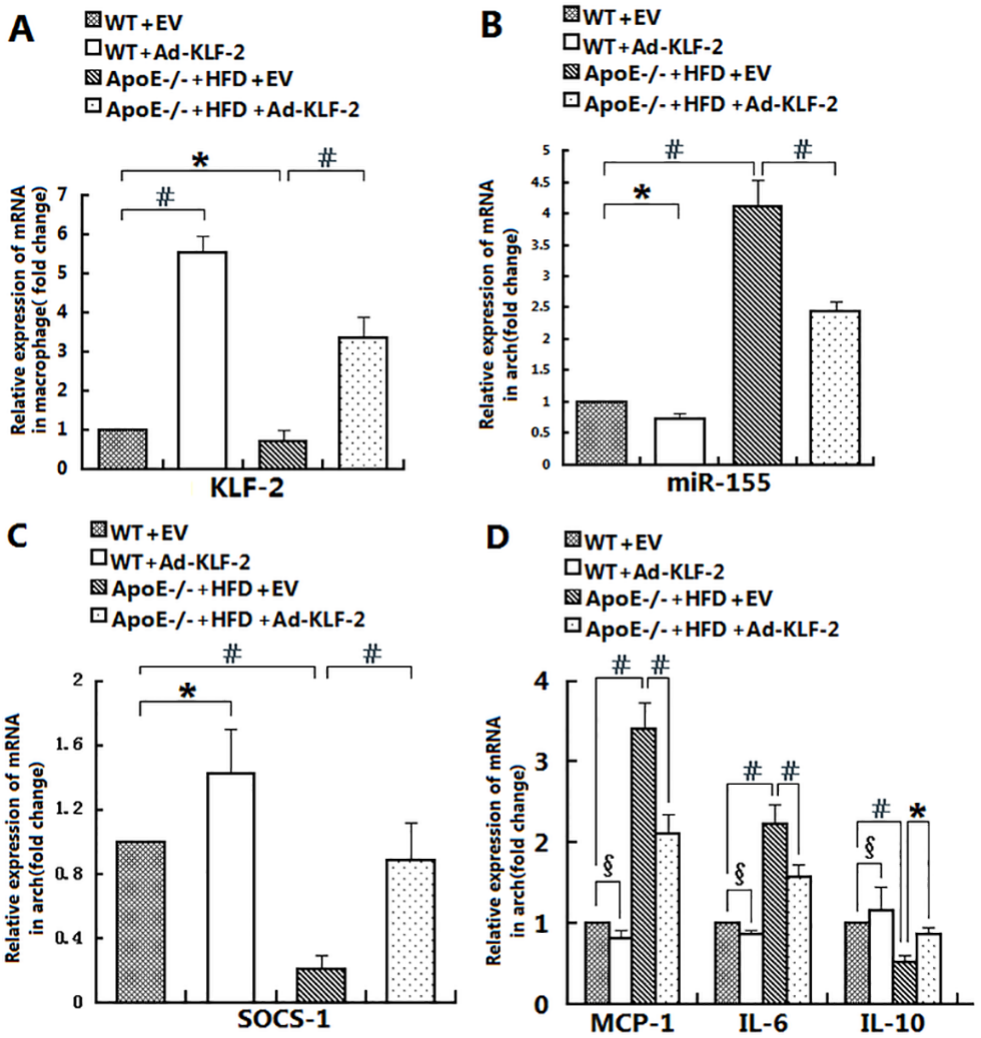
The effects of KLF2 on miR-155/SOCS-1 and cytokine gene expression in pro-inflammatory macrophages. Murine resident peritoneal macrophages were obtained from the four aforementioned groups at the time of euthanization. The mRNA expression levels of KLF2 (A), miR-155 (B), SOCS-1 (C) and cytokines (D) in these cells were determined by RT-PCR (n = 5; #, *, and δ indicate p<0.001, p<0.05 and p>0.05, respectively, for the indicated comparisons).

WT or apoE−/− mice at five weeks of age received one bolus injection of Ad-KLF2 or EV as nonspecific control. After 18 weeks of an atherogenic diet, mice were euthanized, and their aortic arches were harvested for analysis. We then examined the expression of KLF2, miR-155, SOCS-1 and cytokine genes by RT-PCR and Western blotting analysis and the formation of atherosclerotic lesions in the aortic arch sections by staining with H&E, as described previously [16,17,18]. RT-PCR analysis showed that KLF2 was reduced in proatherogenic mice treated with EV compared with WT control mice but was increased in the aortic arches of WT mice +Ad-KLF2 and in apoE-/- mice +HFD +Ad-KLF2 (Fig 4A and p<0.05). Additionally, the aortic arches of apoE-/- +HFD mice expressed higher levels of miR-155 (Fig 4B) but lower levels of SOCS-1 (Fig 4C and 4E) compared with WT mice. KLF2 over-expression significantly decreased the expression of miR-155 (Fig 5B) but increased its target gene SOCS-1 (Fig 4C and 4E) in association with a high level of expression of the inflammatory cytokine genes (MCP-1, IL-6) but a lower level of expression of anti-inflammatory cytokine genes (IL-10) in the aortic arches of apoE-/- mice after 18 weeks of a HFD (Fig 4D and 4F). Examination of the aortic arches (Fig 4H) showed that the atherosclerotic lesions typically developed at lesion-prone sites, such as the lesser curvature of the aortic arch and the ostia of the innominate, common carotid, and the subclavian arteries. There was a marked decrease in the progression of atherosclerotic lesions in apoE-/- mice +HFD treated with Ad-KLF2. Quantitative analysis revealed a significant decrease in the area of the atherosclerotic lesions in Ad-KLF2 treated mice compared with apoE-/- +HFD mice (Fig 4G, 408±11 μm2 versus 598±48 μm2; p<0.05).

### The effect of KLF2 on miR-155/SOCS-1 and the expression of cytokine genes in pro-inflammatory macrophages

We next sought to assess the effect of KLF2 on macrophage function. Murine peritoneal macrophages were obtained from the above four treatment groups. The expression of KLF2, miR-155, SOCS-1 and the production of cytokines in macrophages was determined by RT-PCR. We found a much higher miR-155 (Fig 5B) but lower SOCS-1 (Fig 5C) expression level in the peritoneal macrophages of HFD-treated apoE-/- mice that was associated with a reduced KLF2 (Fig 5A) expression level compared with the macrophages from WT mice. The expression of KLF2 was increased in the peritoneal macrophages of Ad-KLF2- and HFD-treated apoE-/- mice compared with the peritoneal macrophages from HFD-treated apoE-/- mice (Fig 5A), which was accompanied by a significantly decreased miR-155 (Fig 5B) but increased SOCS-1 expression level (Fig 5C). Most importantly, macrophages from Ad-KLF2-treated mice were significantly decreased in their capacity to produce pro-inflammatory cytokines/chemokines (MCP-1, IL-6) compared with macrophages from HFD-treated apoE-/- mice. In contrast, the production of the anti-inflammatory cytokine IL-10 was enhanced (Fig 5D).

## Discussion

The shear-responsive transcription factor KLF2 is a critical regulator of the patterns of endothelial gene expression induced by atheroprotective flow [19]. Of considerable interest, recent evidence indicates that significant interactions exist between KLF2 and miRNAs in endothelial cells. In particular, miR-126 has been reported to be up-regulated by flow in a KLF2-dependent manner in zebrafish embryos [20]. Atheroprotective flow causes a down-regulation of miR-92a in endothelial cells, which in turn elevates KLF2 mRNA [21]. Additionally, miR-143/145 are regulated by KLF2 in endothelial cells and may contribute to the vasculo-protective functions of KLF2 [22].Although KLF2 is known to exert anti-inflammatory effects and inhibit the pro-inflammatory activation of monocytes, whether KLF2 also affects the expression of miRNAs in macrophages and their role in preventing the pro-inflammatory activation of macrophages has remained elusive to date. MiR-155 has been shown to play important roles in immunity and inflammation, particularly in the inflammatory responses of macrophages, implying that it may also be involved in atherogenesis. However, the function of miR-155 in ox-LDL-stimulated inflammation and atherosclerosis remains unclear. Interestingly, the treatment of macrophages with ox-LDL appears to suppress several miRNAs induced after inflammatory stimulation such as miR-146a, miR-155, and miR-21 [23, 24]. However, ox-LDL can also up-regulate miR-125a-5p and miR-155, which reduces the accumulation of lipids and the secretion of cytokine in macrophages [25, 26]. In the current study, we observed that the expression of the inflammation-associated miR-155 was significantly suppressed by KLF2 in unstimulated/ox-LDL-stimulated macrophages (Fig 1A). In contrast, silencing of KLF2 markedly increased miR-155 expression in un-stimulated/ox-LDL-stimulated macrophages (Fig 2A). In agreement with a recently published study [27, 28], we also showed that the exposure of macrophages to ox-LDL led to a marked up-regulation of miR-155 expression, which was positively correlated with the expression of pro-inflammatory cytokines (Fig 1D–1F). Moreover, the effect of recombinant adenovirus-mediated KLF2 significantly attenuated diet-induced atherosclerotic lesion formation in apoE^-/-^ mice and was associated with a significant decrease in the expression of miR-155 and the inflammatory cytokine genes (MCP-1, IL-6) as well as increased expression of the anti-inflammatory cytokine gene (IL-10) in the aortic arches and macrophages of pro-atherogenic mice (Fig 4).

To further confirm a possible role of miR-155 in the KLF2-mediated inhibition of the macrophage inflammatory response, we found by using gain-of-function and loss-of-function approaches that the increased levels of MCP-1 and IL-6 but decreased level of IL-10 in macrophages induced by ox-LDL were further enhanced by over-expression of miR-155. Most interestingly, restoration of miR-155 levels in KLF2 over-expressing macrophages increased MCP-1 and IL-6 but reduced IL-10 expression, indicating that over-expression of miR-155 could partly reverse the suppressive effects of KLF2 on the macrophage inflammatory response induced by ox-LDL (Fig 3A and 3B). Conversely, the increased levels of MCP-1 and IL-6 but decreased level of IL-10 induced in macrophages by ox-LDL were suppressed by inhibition of miR-155. The suppression of miR-155 activity in KLF2-knockdown macrophages with anti-miR-155 significantly overcame the pro-inflammatory properties associated with KLF2 knockdown (Fig 3C and 3D). These results indicate that the KLF2 inhibition of the pro-inflammatory activation of macrophages is at least partly due to KLF2-mediated suppression of the expression of miR-155.

The KLF2 transcription factor has previously been shown to modulate miRNA expression in several cell types. For example, KLF2 binds to the promoter of the miR-143/145 gene cluster to up-regulate the expression of vascular protective genes in endothelial cells [22]. Additionally, KLF2 also mediates the expression of miR-126 in endothelial and glioma cells [20, 29]. Lingrel JB and colleagues recently observed reduced expression of miR-124a and miR-150 in macrophages from myeKlf2^-/-^ mice, thus indicating that KLF2 directly mediates the expression of these two miRNAs in macrophages [30]. To investigate the possibility that KLF2 may directly induce miR-155 transcription, we analyzed the promoter of miR-155 in silico using MatInspector [31]. However, we were unable to detect the presence of the KLF2 transcription binding site in the promoters of miR-155.

Mechanistically, even though a consensus KLF2 binding sequence in the promoters of miR-155 has not yet been identified, the KLF2-regulated transcriptome probably contains a large number of indirect targets as well because KLF2 has been reported to regulate the expression of over a thousand genes [32, 33]. Most of the anti-inflammatory effects of KLF2 (other than from direct eNOS induction) are probably indirect. For instance, KLF2 has been shown to inhibit the transcriptional activity of NF-κB, leading to attenuation of the expression of inflammatory genes [34]. Recent studies have shown that several transcriptional factors, including AP-1, C-myb, and NF-κB, up-regulate the expression of miR-155 in the immune system [35, 36, 37] but the transcriptional repressors of miR-155 remain unknown. Although the molecular basis of the KLF2-mediated inhibition of miR-155 in macrophages remains unknown, our study raises the interesting possibility that the ability of KLF2 to regulate various biological processes may be related to its ability to directly regulate gene transcription activity as well as indirectly modulate cellular miRNA. Because KLF2 and miR-155 play key roles in regulating the function of macrophages in inflammation, additional studies aimed at identifying the relationship between KLF2 expression and miRNA levels in macrophages are warranted.
